# Intestinal Colonization with *Enterococcus faecium* Does Not Influence Pulmonary Defense against *Pseudomonas aeruginosa* in Mice

**DOI:** 10.1371/journal.pone.0006775

**Published:** 2009-08-27

**Authors:** Masja Leendertse, Rob J. L. Willems, Ida A. J. Giebelen, Joris J. T. H. Roelofs, Janetta Top, Marc J. M. Bonten, Tom van der Poll

**Affiliations:** 1 Center for Infection and Immunity Amsterdam, Academic Medical Center, Amsterdam, The Netherlands; 2 Center for Experimental and Molecular Medicine, Academic Medical Center, Amsterdam, The Netherlands; 3 Department of Pathology, Academic Medical Center, Amsterdam, The Netherlands; 4 Department of Medical Microbiology, University Medical Center Utrecht, Utrecht, The Netherlands; 5 Julius Center for Health Studies and Primary Care, University Medical Center Utrecht, Utrecht, The Netherlands; Hannover School of Medicine, Germany

## Abstract

**Background:**

Enterococci, and especially multiresistant *Enterococcus faecium*, are increasingly found colonizing hospitalized patients. This increased prevalence of colonization is not only associated with an increased prevalence of infections caused by enterococci, but also by infections with other nosocomial pathogens. In this study we investigated the causality of this observed relationship, by determining the influence of intestinal colonization with *E. faecium* on pulmonary defense against *Pseudomonas aeruginosa*.

**Methodology/Principal Findings:**

Three groups of mice were tested; 2 groups of mice were pre-treated with vancomycin, of which one group was subsequently treated by oral gavage of vancomycin-resistant *E. faecium* (VRE). The third group did not receive any pre-treatment. *P. aeruginosa* pneumonia was induced in all mice. Vancomycin treatment resulted in intestinal gram-negative bacterial overgrowth and VRE treatment resulted in colonization throughout the intestines. All 3 groups of mice were able to clear *P. aeruginosa* from the lungs and circulation, with comparable lung cytokine responses and lung damage. Mice treated with vancomycin without VRE colonization displayed modestly increased plasma levels of TNF-α and IL-10.

**Conclusion:**

Overgrowth of *E. faecium* and/or gram-negative bacteria does not impact importantly on pulmonary defense against *P. aeruginosa* pneumonia.

## Introduction

Hospitalized patients become increasingly colonized with multiresistant *Enterococcus faecium*. This is mainly observed in patients treated with antibiotics not effective against *E. faecium* on nephrology, ICU, hematology and transplantation wards [1], [2]. Colonization with this opportunistic pathogen not only has been associated with an increased incidence of infections with *E. faecium* [3], [4], but also with other nosocomial pathogens including multiresistant gram-negative bacilli [5], [6]. At present it is unclear whether this association between enterococcal colonization and the increased incidence of nosocomial infections bears a causal relationship or is reflective of the immune suppression commonly observed in hospitalized patients [7]. Previous experimental studies have supported the first possibility, showing that alterations in the human gastrointestinal tract microbiota can alter the host immune response and that intestinal bacterial overgrowth, even in the absence of tissue injury or gut ischemia, can impair systemic immune responsiveness [8], [9]. Indeed, Marshall et al. [9] demonstrated that oral administration of heat killed *Pseudomonas aeruginosa* for 21 days reduced the clearance of a subcutaneous *Staphylococcus aureus* abscess and suppressed nonspecific immunity, as measured by the delayed-type hypersensitivity response, in rats. In line, Deitch et al. [10] reported a decreased *in vitro* lymphocyte mitogenic response and a diminished ability to control a *S. aureus* abscess in mice orally treated with *Escherichia coli*.

*Pseudomonas aeruginosa* is a common causative pathogen in nosocomial pneumonia and lower respiratory tract infections are the leading cause of death from nosocomial infections [11]. In light of the association between enterococcal colonization and infection with other nosocomial pathogens, and the experimental evidence that alterations in the gut flora may impact on host immunity, we here investigated the causality of this observed relationship and demonstrate that intestinal overgrowth with *E. faecium* does not influences the pulmonary immune response to *P. aeruginosa*.

## Materials and Methods

### Mice

Specific pathogen-free 10-wk-old female wildtype C57BL/6 mice were purchased from Harlan Sprague-Dawley (Horst, The Netherlands). The animals were housed in rooms with a controlled temperature and a 12-h light-dark cycle. They were acclimatized for 1 week prior to usage, and received standard rodent chow and water ad libitum. The Animal Care and Use Committee of the University of Amsterdam approved all experiments.

### Bacterial strains

A vancomycin resistant *E. faecium* (VRE) strain, E155, was used in all experiments. This clinical isolate from the Cook County Hospital, Chicago, IL, belongs to a genetic subpopulation of hospital-associated *E. faecium*, currently labeled clonal complex-17 (CC17) that is responsible for the worldwide emergence of nosocomial multiresistant *E. faecium*. CC17 is characterized by high-level quinolon resistance, ampicillin resistance and a recently identified pathogenicity island, containing the variant *esp* gene [12]. For all experiments the bacteria were grown overnight on agar sheep blood (BA) plates and then grown for approximately 3.5 hours in Todd-Hewitt (TH) broth (Difco, Detroit, MI) to midlogarithmic phase at 37°C, while shaking. *P. aeruginosa* (strain PA01) was grown to mid-logarithmic phase in Luria Bertani medium (Difco) for 6 h at 37°C.

### Experimental design

A mouse model of VRE gastrointestinal colonization was developed according to the model described by Whitman et al. [13]. Three groups of mice (N = 8 per group) were studied. In group 1 VRE colonization was established by administering oral vancomycin (250 µg/ml) in drinking water for 5 days before gastric inoculation of 10^7^ CFU *E. faecium* suspended in 300 µl TH medium; control mice (group 2) were treated with oral vancomycin but received TH medium without *E. faecium*. Vancomycin was continued during the entire experiment in both groups 1 and 2. An additional control group (group 3) was included without prior vancomycin treatment or *E. faecium* colonization. For quantification of intestinal VRE fresh stool was plated on Slanetz-Bertley (SB) agar plates (Oxoid, Badhoevedorp, The Netherlands), supplemented with (6 µg/ml) vancomycin. Additionally, stool samples were plated onto SB agar plates without vancomycin to enumerate total enterococcal load, and BA, MacConkey (McC) (Difco, Detroit, MI) and colistin nalidixic acid (CNA) (BD, Breda, The Netherlands) agar plates for quantification of total aerobic, facultative gram-negative and gram-positive bacteria respectively. After 14 days of *E. faecium* colonization some mice were sacrificed to check for *E. faecium* colonization throughout the intestines, bacterial translocation to mesenteric lymph nodes (MLN) and histopathology of the intestines directly prior to induction of pneumonia. Pneumonia was induced 19 days after initiation of vancomycin treatment (groups 1 and 2) and 14 days after *E. faecium* colonization (group 1). To induce pneumonia mice were lightly anaesthetized with inhaled isoflurane (Abbott, Queensborough, Kent, UK) and an inoculum of 5×10^6^ CFU *P. aeruginosa* suspended in 50 µl saline was administered intranasally as described [14], [15].

### Preparation of blood samples and homogenates

Mice were sacrificed 6, 24 or 48 hours after inducing pneumonia. They were anesthetized with Hypnorm (Janssen Pharmaceutica, Beerse, Belgium) and midazolam (Roche, Mijdrecht, The Netherlands). Blood was drawn by cardiac puncture, transferred to heparin-gel vacutainer tubes and immediately placed on ice. Then MLN, lungs, jejunum, cecum and colon were harvested and homogenized in 4 volumes of sterile saline with a tissue homogenizer (Biospect Products, Bartlesville, UK), which was carefully cleaned and disinfected with 70% ethanol after each homogenization. Blood was spun down and plasma was collected and frozen at -20°C until assayed. For lung and intestinal cytokine measurements lung homogenates were lysed in 1 volume of lysis buffer (300 mM NaCl, 15 mM Tris, 2 mM mgClH2O, 2 mM Triton X-100, pepstatin A, leupeptin, and aprotinine (20 ng/ml), pH 7.4) on ice for 30 minutes and spun down. Supernatants were frozen at −20°C until assayed.

### Determination of bacterial outgrowth

Serial 10-fold dilutions were made of intestinal contents and each sample of blood, lung and MLN homogenates in sterile saline and 50 µl of each dilution was plated onto BA plates; intestinal contents and MLN were also plated on McC, CNA and SB agar plates. The plates were incubated at 37°C under 5% CO_2_, and CFU were counted after 20 hours, or 44 hours for SB plates, and corrected for the dilution factor.

### Typing bacteria by multiple-locus variable-number tandem repeat analysis

Multiple-locus variable-number tandem repeat (VNTR) analysis (MLVA) was used to identify VRE from stool as *E. faecium* strain E155. The MLVA typing was performed as described previously [16] (www.mlva.umcutrecht.nl), with the following minor modifications: PCR programs for VNTR-8 and VNTR-9 were similar to VNTR-7 and VNTR-10, while for VNTR-2 the extension time was prolonged to 4 minutes instead of 30 s, at 72°C.

### Cytokine measurements

Tumor necrosis factor (TNF)-α, interleukin (IL)-6, IL-10 and monocyte chemoattractant protein (MCP)-1 were measured in homogenates of lung and intestines and plasma by using a commercially available cytometric bead array multiplex assay (BD Biosciences, San Jose, CA) in accordance with the manufacturer's recommendations.

### Statistical analysis

All data are expressed as mean±SEM. Differences between groups were calculated by Mann-Whitney *U* test. For all analysis GraphPad Prism version 4 (GraphPad Software, San Diego, CA) was used. A *p*-value <0.05 was considered statistically significant.

## Results

### Intestinal *E. faecium* colonization

The intestinal track of mice receiving vancomycin in combination with oral *E. faecium* was successfully colonized with VRE. In this experimental group 1, high VRE loads were cultured from jejunum, cecum and colon directly before induction of pneumonia (Table 1). These colonies were confirmed *E. faecium* strain E155 by MLVA typing. VRE could not be cultured from the intestines of mice from experimental groups 2 or 3. Enterococci cultured from stools of control mice, with or without vancomycin treatment, were identified as *Enterococcus faecalis*. In vancomycin treated mice (groups 1 and 2) the gram-negative bacterial load in stool was almost 1000-fold higher than in untreated mice (group 3) (Table 2). Similar shifts in the fecal flora in vancomycin treated mice were seen in jejunum, cecum and colon (data not shown). An increase was seen in bacterial translocation in the vancomycin treated mice; 2 out of 8 untreated mice showed positive cultures of MLN, harboring both gram-negative and gram-positive bacteria. In both groups of vancomycin treated mice 5 out of 8 MLN cultured positive. Three of the VRE colonized mice had VRE positive MLN. Neither systemic dissemination of bacteria nor an increase in bacterial translocation after induction of pneumonia was detected. Upon histopathological analysis of jejunum, cecum and colon tissue the epithelial cell lining appeared intact and no remarkable pathology was seen (data not shown). No increases in cytokine levels were measured in the intestinal homogenates (data not shown).

**Table 1:** Fecal VRE counts in different intestinal compartments of VRE colonized mice.

Intestinal compartment	VRE counts/gram of intestinal contents
Jejunum	2,1±0.1×10^6^
Cecum	6,0±0.9×10^8^
Colon	9,2±0.6×10^8^

Mice were treated with vancomycin (250 µg/ml) in drinking water for 19 days; VRE was administered by gastric inoculation (10^7^ CFU) 5 days after the initiation of vancomycin treatment. Specimens were obtained 14 days after VRE instillation. Data are means±SE of 8 mice.

**Table 2:** Total enterococcal and aerobic gram-negative count in fecal pellets.

Treatment groups	Total enterococcal count/gram feces	Total gram-negative count/gram feces
Vancomycin and VRE	4.0±1.3×10^9^ ***/†	1.2±0.1×10^9^ ***
Vancomycin	3.0±0.4×10^5^ ***	1.4±0.4×10^9^ ***
Untreated	5.5±0.9×10^7^	3.9±0.2×10^6^

Two groups of mice were treated with vancomycin for 19 days; in one of these, VRE was administered by gastric inoculation (10^7^ CFU) 5 days after the initiation of vancomycin treatment. A third group of mice did not receive vancomycin or VRE. Enterococci, isolated from untreated mice and from mice treated with vancomycin only, were identified as *E. faecalis* (i.e. normal inhabitants of the intestines).

*** p<0.001 compared to untreated mice, †p<0.001 compared to only vancomycin treated mice (without VRE). Data are means±SE of 8 mice per group.

### Intestinal *E. faecium* colonization does not impact on the clearance of *P. aeruginosa* from the lungs

**Figure 1:**
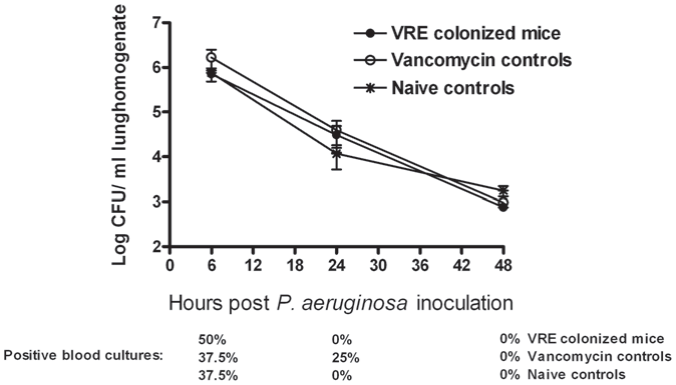
Comparable *P. aeruginosa* clearance from lungs in all treatment groups. Three groups of mice were intranasally inoculated with *P. aeruginosa* after different pre-treatment schedules. Two groups of mice were treated with vancomycin for 19 days; in one of these VRE was administered by gastric inoculation (10^7^ CFU) 5 days after the initiation of vancomycin treatment (VRE colonized mice), while in the other group no VRE was administered (Vancomycin controls). A third group of mice did not receive vancomycin or VRE (Naïve controls). Percentages of positive blood cultures at different time points are depicted below the graphic. Data are mean±SEM of 8 mice per group at each time point.

After 14 days of colonization and 19 days of vancomycin treatment, mice were intranasally inoculated with 5×10^6^ CFU *P. aeruginosa*. Mice were sacrificed 6, 24 and 48 hours after induction of pneumonia. In all mice a gradual decrease in the number of *P. aeruginosa* CFU was measured in lungs without differences between VRE colonized mice, mice with intestinal gram-negative overgrowth due to vancomycin treatment but without VRE colonization, or mice with normal intestinal flora (Fig. 1). Six hours after the induction of pneumonia 50% of VRE colonized and 37.5% of mice in the two other groups had positive blood cultures with *P. aeruginosa* (Fig. 1). After 24 hours 25% of mice treated with vancomycin but without VRE colonization still had positive blood cultures, although only low amounts of *P. aeruginosa* were cultured. Forty eight hours after the infection none of the mice were bacteremic.

### Intestinal *E. faecium* colonization does not influence the host response to *P. aeruginosa* pneumonia

**Figure 2:**
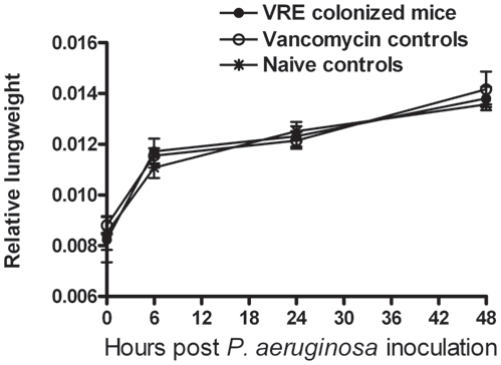
Comparable increase in relative lung weight (mg whole lung/gram mouse) during *P. aeruginosa* pneumonia in all 3 groups. Three groups of mice were intranasally inoculated with *P. aeruginosa*, after different pre-treatment schedules. Two groups of mice were treated with vancomycin for 19 days; in one of these VRE was administered by gastric inoculation (10^7^ CFU) 5 days after the initiation of vancomycin treatment (VRE colonized mice), while in the other group no VRE was administered (Vancomycin controls). A third group of mice did not receive vancomycin or VRE (Naïve controls). Data are mean±SEM of 8 mice per group at each time point.

**Figure 3:**
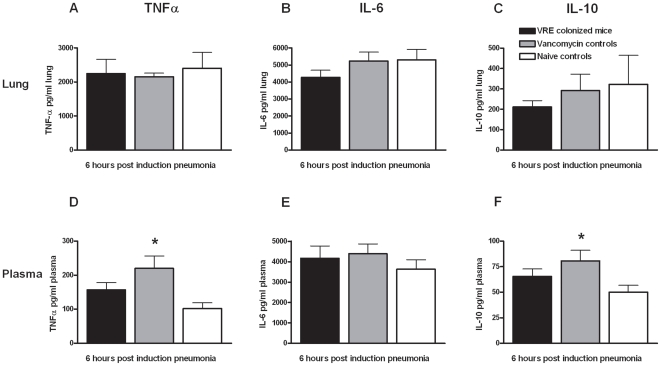
Cytokine levels 6 hours after induction *P. aeruginosa* pneumonia. TNF-α (A and D), IL-6 (B and E) and IL-10 (C and F) levels in lung and plasma of mice, 6 hours after intranasal inoculation with *P. aeruginosa*. Two groups of mice were treated with vancomycin for 19 days; in one of these VRE was administered by gastric inoculation (10^7^ CFU) 5 days after the initiation of vancomycin treatment (VRE colonized mice), while in the other group no VRE was administered (Vancomycin controls). A third group of mice did not receive vancomycin or VRE (Naïve controls). Data are mean±SEM of 8 mice per group; * *p*<0.05 versus mice without vancomycin treatment or VRE colonization.

*Pseudomonas* pneumonia was associated with an increase in the relative lung weights in all mice, indicative for inflammation and edema (Fig. 2) [14]. Neither VRE colonization nor gram-negative intestinal overgrowth influenced this response. In line, no differences were seen between the three experimental groups with regard to histopathology: the lungs of all mice showed interstitial inflammation, edema and pleuritis. Neither VRE colonization nor intestinal gram-negative overgrowth impacted on the local cytokine response to *Pseudomonas* pneumonia. Indeed, no differences were found between groups with regard to the pulmonary levels of TNF-α, IL-6, IL-10 or MCP-1 at either 6, 24 or 48 hours after induction of pneumonia (shown for TNF-α, IL-6 and IL-10 at 6 hours in Fig. 3A–C). Mice treated with vancomycin but not colonized with VRE had modestly but statistically significantly higher plasma TNF-α and IL-10 levels 6 hours after the start of the infection (Fig. 3D–F). Plasma cytokine concentrations were similar in all groups at 24 and 48 hours after induction of pneumonia (data not shown).

## Discussion

In this study we show that intestinal colonization with a multidrug- and vancomycin-resistant, clinical isolate of *E. faecium* (VRE) does not influence the host response to *P. aeruginosa* pneumonia. Colonization with VRE did not result in alterations in the clearance of *Pseudomonas* from the lungs, differences in local or systemic inflammatory cytokine responses or in pulmonary pathology. Additionally, intestinal gram-negative bacterial overgrowth secondary to vancomycin treatment, although associated with slightly higher plasma TNF-α and IL-10 concentrations shortly after induction of pneumonia, did neither influence host defense or lung pathology during *P. aeruginosa* pneumonia.

Colonization with multi- and vancomycin- resistant *E. faecium* is a growing problem in the hospital setting. Colonization with this opportunistic pathogen has been associated with an increased incidence of infections with *E. faecium* and increased mortality [3], [4], [17], [18]. Additionally, colonization with VRE has been associated with infections by other nosocomial pathogens including multiresistant gram-negative bacilli [5], [6]. Many risk factors for VRE colonization and infection are shared by other nosocomial pathogens, among which multidrug-resistant gram-negative bacteria. These factors include preceding antibiotic exposure, increased severity of illness and increased length of hospital stay [19], [20]. One explanation of the association between enterococcal colonization and the increased incidence of nosocomial infections could be a common manifestation of the immune suppression frequently observed in hospitalized patients [7]. Furthermore, patients with an impaired immune system, such as hematology and transplant patients, are at the highest risk for acquiring enterococcal infections [21]. In the current study we sought to obtain insight into the validity of an alternative explanation, i.e. that colonization of the intestinal tract with VRE impairs the immune response to *P. aeruginosa*, a well known causative agent of nosocomial pneumonia. The most important result of this study was that the response to respiratory tract infection with *Pseudomonas* was virtually identical, despite major differences in the intestinal flora, for mice colonized and not-colonized with *E. faecium*.

Previous experimental studies have shown that alterations in the flora of the gastrointestinal tract can alter the host immune response and that intestinal bacterial overgrowth, even in the absence of tissue injury or gut ischemia, can impair systemic immune responsiveness [8], [9]. Indeed, oral administration of *P. aeruginosa* or *E. coli* impaired host defense against a subcutaneous *S. aureus* abscess [9], [10], whereas oral gavage of killed *P. aeruginosa* or *Candida albicans* produced significant depression of the delayed-type hypersensitivity response [9]. In another study [22], peritonitis was shown to be accompanied by massive small-bowel overgrowth with *E. coli* in rats, which contributed to suppression of systemic immunity. Furthermore, mice orally treated with *E. coli* demonstrated a decreased in vitro lymphocyte mitogenic response [10] and starvation and protein malnutrition disrupted the normal indigenous gastrointestinal tract microflora in mice, which was found to impair host antibacterial defenses [23]. In other studies it was demonstrated that alteration in the gut microflora modulates the systemic cytokine response to hemorrhagic shock, with intestinal bacterial overgrowth of monoassociated *E. coli* leading to the greatest increase in plasma IL-6 and TNF-α levels [24], [25]. Of note, this latter finding is in line with our current data revealing modestly elevated plasma TNF-α and IL-10 concentrations 6 hours after induction of *Pseudomonas* pneumonia in mice with intestinal gram-negative overgrowth without VRE colonization.

In this study, we did not observe an influence of colonization with *E. faecium* on the innate immune response to *P. aeruginosa* pneumonia. *E. faecium*, in addition to the most frequently used genera *Lactobacillus* and *Bifidobacterium*, is one of the lactic acid bacteria (LAB), referred to as probiotics [26]. Probiotics are non-pathogenic bacteria that are used as functional food components with claimed health-promoting effects. Several studies have been performed to investigate the role of intestinal *E. faecium* on the immune system in young animals. In two studies performed by Scharek et al. [27], [28] the influence of intestinal colonization by a non-pathogenic *E. faecium* strain, SF68, on the immune system was tested in piglets. They found a reduction in the systemic humoral immunity (total serum IgG) and a reduction of CD8+ cells in the intestinal epithelium of the upper jejunum. Contrarily, Benyacoub et al. [29] found a stimulatory effect of colonization by SF68 on the immune system, on mucosal and systemic IgA and IgG levels in dogs. In kittens, Veir et al. [30] found no influence of *E. faecium* colonization on the specific or nonspecific immune parameters, besides an increased percentage of CD4+ cells. Recently, we found mice first colonized with VRE and subsequently undergoing intestinal perforation by cecal ligation and puncture (CLP) had improved infectious and inflammatory outcomes of polymicrobial peritonitis compared to mice undergoing CLP without the presence of *E. faecium* (31). The mechanism by which VRE colonization and/or infection influenced the host response to polymicrobial peritonitis remains to be established.

Colonization and infection with multiresistant *E. faecium* have been associated with infections by other nosocomial and multiresistant pathogens. In this study we investigated the influence of profound intestinal alterations on the pulmonary response to *P. aeruginosa*. We showed that colonization with VRE and/or intestinal overgrowth with gram-negative bacteria does not impact importantly on the innate immune response to *P. aeruginosa* pneumonia. These data argue against the possibility that increased enterococcal colonization increases the risk of infections by other nosocomial pathogens by impairing the immune response of the host. Furthermore, our data also show that alterations in the intestinal flora do not influence host defense against a common nosocomial respiratory pathogen.
